# High-throughput antibody screening from complex matrices using intact protein electrospray mass spectrometry

**DOI:** 10.1073/pnas.1917383117

**Published:** 2020-04-23

**Authors:** William S. Sawyer, Neha Srikumar, Joseph Carver, Phillip Y. Chu, Amy Shen, Ankai Xu, Ambrose J. Williams, Christoph Spiess, Cong Wu, Yichin Liu, John C. Tran

**Affiliations:** ^a^Department of Biochemical and Cellular Pharmacology, Genentech, Inc., South San Francisco, CA 94080;; ^b^Department of Cell Culture and Bioprocess Operations, Genentech, Inc., South San Francisco, CA 94080;; ^c^Department of Purification Development, Genentech, Inc., South San Francisco, CA 94080;; ^d^Department of Antibody Engineering, Genentech, Inc., South San Francisco, CA 94080

**Keywords:** antibody screening, high throughput, intact protein mass spectrometry, electrospray

## Abstract

Toward the goal of increasing the throughput of high-resolution mass characterization of intact antibodies, we developed a RapidFire–mass spectrometry (MS) assay using electrospray ionization. We achieved unprecedented screening throughput as fast as 15 s/sample, which is an order of magnitude improvement over conventional liquid chromatography (LC)-MS approaches. The screening enabled intact mass determination as accurate as 7 ppm with baseline resolution at the glycoform level for intact antibodies. We utilized this assay to characterize and perform relative quantitation of antibody species from 248 samples of 62 different cell line clones at four time points in 2 h using RapidFire–time-of-flight MS screening. The screening enabled selection of clones with the highest purity of bispecific antibody production and the results significantly correlated with conventional LC-MS results. In addition, analyzing antibodies from a complex plasma sample using affinity-RapidFire-MS was also demonstrated and qualified. In summary, the platform affords high-throughput analyses of antibodies, including bispecific antibodies and potential mispaired side products, in cell culture media, or other complex matrices.

## Significance

While electrospray ionization–mass spectrometry (ESI-MS) provides higher resolution for larger proteins, the conventional liquid chromatography (LC)-MS method suffers from low throughput. Our described RapidFire-MS workflow demonstrated unprecedented screening throughput as fast as 15 s/sample, a 10-fold improvement over conventional LC-MS approaches. The screening enabled selection of clones with the highest purity of bispecific antibody production with intact masses as accurate as 7 ppm with baseline resolution at the glycoform level in samples as complex as plasma sample. The utility of the method can be expanded to many other applications that can exploit the advantages of high-throughput intact protein MS analyses including but not limited to pharmacokinetic analyses, enzymatic screening, biotransformation characterization, and quality control screening.

Unambiguous characterization of analytes from complex matrices with high content information in a label-free format continues to expand the application of mass spectrometry (MS) in drug discovery (1–3). With the need for high accuracy, sensitivity, and selectivity, the rapidly improving MS instrumentations are emerging at the forefront of analyzing analytes with limited alternative assays for biologics developability (4–6), biotransformation, and high-throughput screening (HTS). For ultra-high–throughput screening, matrix-assisted laser desorption/ionization (MALDI) is amenable to speeds >100,000 samples per day (7–11). Through incorporating self-assembled monolayers for MALDI–time-of-flight (TOF) (SAMDI) technology (12–14), it is also possible to infer small molecule noncovalent hit identification from MALDI-MS detection under native conditions. While such MALDI-MS approaches have the capability for screening small molecule and peptide analytes, larger molecular weight proteins suffer from limited mass resolution and quantitative challenges. Alternatively, electrospray ionization MS (ESI-MS) can provide isotopic resolution for molecules as large as antibodies (15). Although modern mass spectrometers can scan as rapidly as tens of microseconds, high-throughput antibody analyses using ESI-MS is challenged by the relatively low ion sampling rate from chromatographic elution coupled to MS. For rapid sampling, Agilent RapidFire MS (RF-MS) utilizes a rapid trap and elute strategy to enable desalting and ion sampling coupled to a MS ion source. RF-MS has been shown to afford HTS triage for small molecules and proteins <30 kDa from biochemical buffers as fast as 15 s as opposed to minutes observed with conventional chromatography (16). Alternatively, a recently developed SampleStream device (17) can also afford rapid ion sampling through a process similar to asymmetric flow field flow fractionation. However, high-throughput ESI screening of antibody mixtures (>140 kDa) from complex matrices at the intact level, an unmet need in biopharmaceutical drug discovery, has not been reported.

**Fig. 1.**
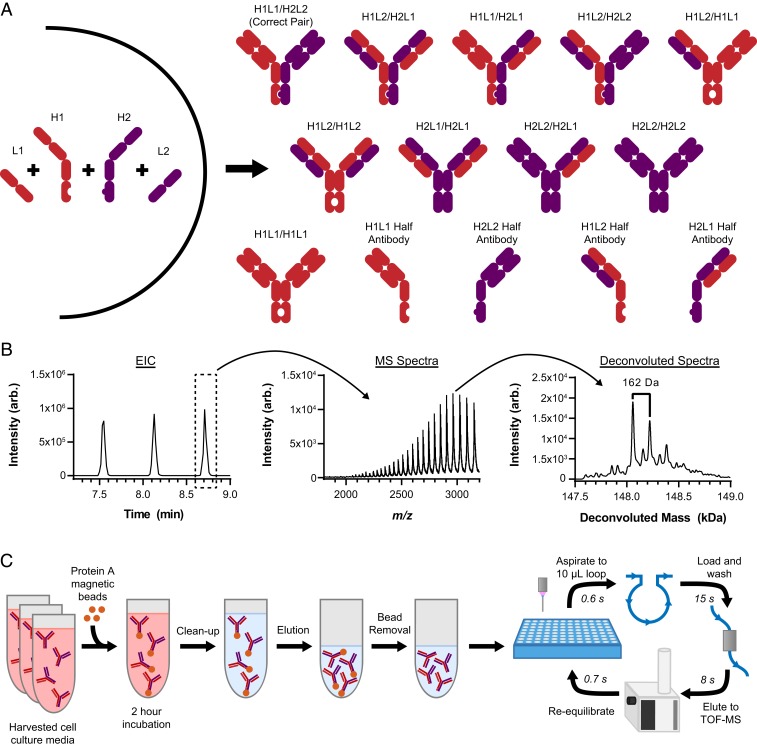
Description of the affinity capture RapidFire mass spectrometry workflow. (*A*) During production, bispecific antibodies generated in a single cell can assemble in different combinations of heavy chains (HC) and light chains (LC). Heterodimerization of the HCs was facilitated using the knobs-into-holes mutations in the Fc region and v7 charge pair mutations for cognate LC pairing (22). In addition to the amount of correctly paired bispecific antibodies, the presence of 13 possible side products needs to be quantified to identify the best single cell clones. (*B*) Extracted ion chromatogram (EIC) of trastuzumab standard revealed the rapid ion sampling afforded by the RF system. Each peak on the EIC represents a separate injection. Online coupling of RF to TOF affords raw MS data acquisition revealing the charge state envelope for trastuzumab. Offline data processing through deconvolution of the MS charge states provided the neutral intact mass of trastuzumab and the corresponding partially resolved glycosylation peaks (∼160 Da). (*C*) Schematic depicting the workflow involving affinity capture for cell culture media and plasma samples containing bispecific antibodies purified using protein A magnetic beads prior to introducing to the RF-MS instrument.

The major impact of antibody therapeutics in treating diseases, such as cancer, has led to the rise of many new format large molecules (18), such as bispecific antibodies (19, 20), which enables the binding of multiple antigens that are useful for many therapeutic applications. Here, we report a robust method for high-throughput screening to characterize bispecific immunoglobulin G (IgG) (BsIgG) generated by coexpressing two different light and heavy chains in a single host cell (21, 22). While this strategy of generating BsIgG in a single cell is more efficient and economical versus using multiple host cells, unwanted mispaired IgG species (e.g., light chain mispairs and half-antibodies) can be produced in addition to the desired BsIgG (Fig. 1*A*). To achieve the throughput required for screening thousands of cell line clones to identify clones with high titer and correct heterodimers, traditional immunoassays such as enzyme-linked immunosorbent assay (ELISA) were typically employed as a primary screen. However, using these assays, each BsIgG campaign requires significant assay development and validation effort up front when using these traditional analytical methods. While MS is routinely used to characterize the quality of the final purified bispecific antibody (23–25), throughput remains a limitation for screening applications. Using the RF-MS assay we can rapidly screen hundreds, and in principle, thousands of cell line clones through characterizing and quantitating bispecific antibodies to identify the clone that generates the desired BsIgG with minimal mispaired side products. Unlike with the ELISA detection traditionally used for BsIgG screening, the multiplexed RF-MS detection capability affords comprehensive identification of multiple species in a single label-free experiment.

## Results and Discussion

### RF-MS Instrument Optimization.

Since RF-MS has not been previously reported for relatively large proteins, we first initiated experiments on optimizing the RF instrument for increased sensitivity. Large macromolecule analysis using solid-phase extraction poses challenges including stationary phase carryover and recovery that must be considered. Trastuzumab was selected for method optimization because it has been well characterized by intact TOF-MS instrumentation, allowing for benchmark comparisons against RF-TOF analysis. We evaluated different stationary phase cartridges for trastuzumab (*SI Appendix*, Fig. S1) and observed that using C4 stationary phase resulted in the highest signal intensities, whereas pore sizes of 1,000 or 4,000 Å did not contribute to significant differences. For the stationary phase tested, the higher resin volume of 10 µL versus 4 µL provided higher intensities for concentrations tested between 50 and 5,000 nM. However, since peak splitting was observed with the 10 µL resins at the flow rates tested, we opted to use the lower capacity 4 µL volume resin (*SI Appendix*, Fig. S2). After identifying the optimal column cartridge (4 µL C4 4,000 Å resin), we attempted to minimize the constraints limiting the highest throughput that can be afforded. For trastuzumab (Fig. 1*B*), the duty cycle between injections (sample and blank) was as fast as 15 s (coefficient of variation CV < 5%). We have also tested other proteins including carbonic anhydrase (*SI Appendix*, Fig. S3) and bovine serum albumin with very similar throughput. After further optimization for sensitivity, we determined that a loading of 0.2 mL/min (15 s) and elution of 0.4 mL/min (8 s) provided the highest intensities for trastuzumab resulting in a 25-s cycle time between injections, including sampling and re-equilibration, which corresponds to a throughput of ∼3,400 samples per day. Sample carryover using this method is <4% for antibodies in the 50 to 5,000 nM concentration range.

### Incorporating Affinity Capture to RF-MS for Bispecific Characterization on Spiked Standards in Cell Culture Fluid.

**Fig. 2.**
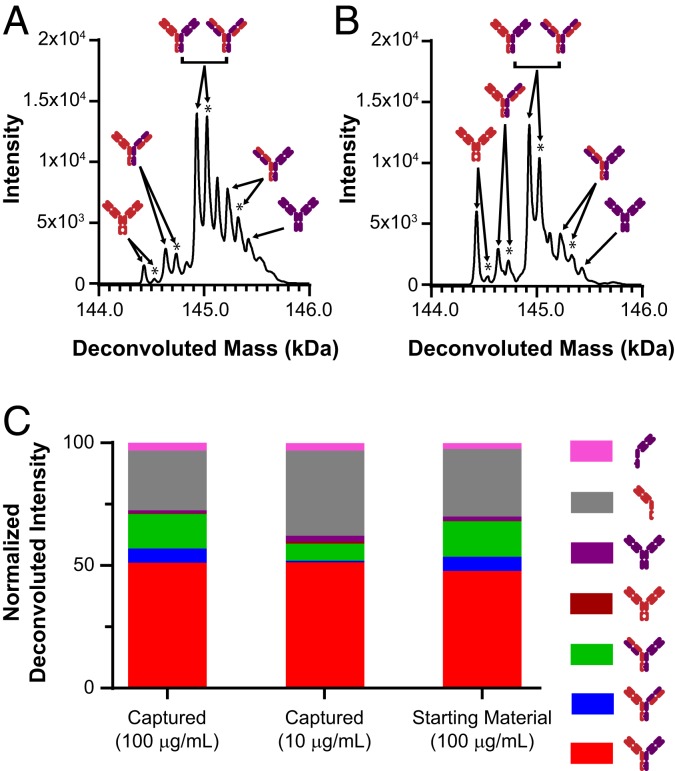
Comparing affinity capture bias from spiked in samples versus starting material. Purified bispecific antibodies were spiked into blank cell media at 100 µg/mL and 10 µg/mL and affinity captured using the RF-MS workflow described in Fig. 1*C*. Deconvoluted spectra showed a similar composition of bispecific antibody pairings in both the protein A captured material (*A*) and the starting material (*B*). Peaks marked by an asterisk denote phosphate adducts. (*C*) The percent composition of correctly paired bispecific antibodies (spiked in cell media) after protein A capture compared to purified starting material (spiked in buffer) yielded 51.1% and 48.7%, respectively (red bars). A similar profile (51.3% correctly paired) was observed on protein concentrations as low as 10 µg/mL for protein A captured antibodies from cell media.

Using the optimized RF-MS methods, we first attempted to analyze the bispecific antibodies from harvested cell culture fluid (HCCF) without offline sample cleanup. In result, low sensitivity and high background, attributed to signal interference from the media matrix was observed. To reduce the sample complexity, an affinity capture procedure using protein A-immobilized magnetic beads were incorporated into the workflow (Fig. 1*C*). The spectral quality of a purified BsIgG spiked into buffer versus blank cell media undergoing the affinity capture process showed very similar MS features from the raw data and processed deconvoluted data (Fig. 2 *A* and *B*). Comprehensive interpretation of the deconvoluted spectra revealed similar compositions of identified species at both 10 µg/mL and 100 µg/mL, suggesting minimal bias from the enrichment procedure (Fig. 2*C*). The signal intensity of the 10 µg/mL concentration was similar to what was obtained from the actual seed train (day 0) cell media samples (*SI Appendix*, Fig. S4).

### Increasing MS Resolution from RF Coupled to FTMS Platform.

A closer inspection of the processed deconvoluted MS spectra revealed the presence of half-antibodies, phosphate adducts, and C-terminal glycine cleavage (Fig. 2*A*). It should be noted that there is likely an MS response bias between the different species and therefore accurate quantitation by peak intensities among different masses is not possible without calibration curves. Therefore, only relative quantitation among the same masses across samples was performed. The TOF-MS instrument sufficiently resolved and differentiated the various antibody species from mispaired chain pairing (Fig. 2 *A* and *B*). Further, the TOF instrument was also able to partially resolve the species with phosphate adducts (+98 Da). To expand the capability of the platform for samples requiring enhanced resolution, coupling RF to Fourier transform MS (FTMS) was demonstrated on trastuzumab (*SI Appendix*, Fig. S5). In contrast to the TOF mass analyzer (Fig. 1*B*), FTMS can easily baseline resolve intact antibody glycoforms, with mass differences of ∼160 Da within 7 ppm mass accuracy.

### Single Cell Clone Selection Screening of Bispecific Antibodies Using RF-MS.

We ran a pilot study using 40 samples from eight different BsIgG-producing transfection pools at five time points each (*SI Appendix*, Fig. S6). Negative controls included a nonproducing cell line as well as blank cell growth media. The final time point for each pool was also evaluated by liquid chromatography (LC)-MS for comparison against RF-MS data (*SI Appendix*, Fig. S7). Deconvoluted RF-MS spectra for day 14 samples indicated that the primary impurity in low-performing pools (1, 2, 5, 6) was half-antibody. Comparing these pools against the high-performing pools (3, 4, 7, 8) showed a decrease in overall intensity of correctly paired bispecific antibodies as well as an increase in the overall intensity of half-antibodies, which both contribute to the lower percent of correct pair matching.

**Fig. 3.**
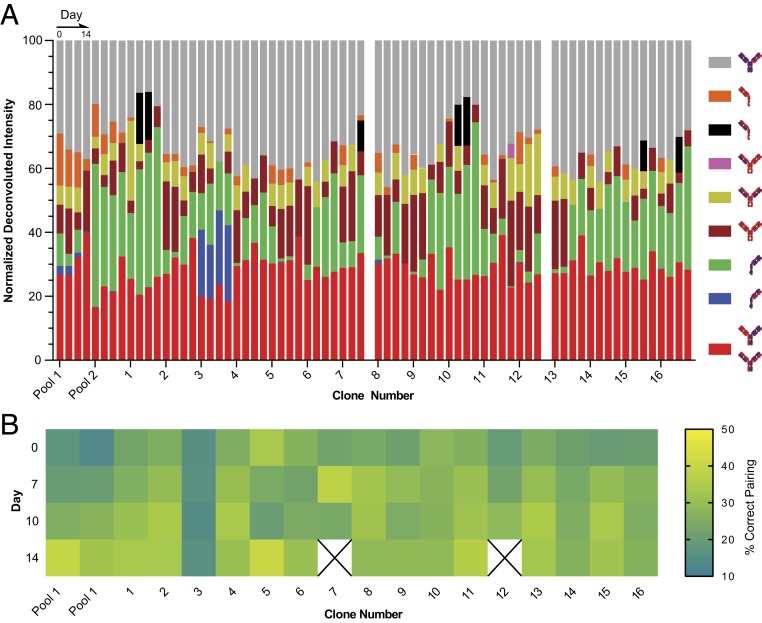
Results from the bispecific cell line clone selection screening. Composition analysis of 2 transfection pools and 16 single cell clones collected at days 0, 7, 10, and 14 with results generated from the workflow illustrated in Fig. 1*C* (*A*) Deconvoluted intensities of the different combinations of bispecific antibody HC and LC chain conjugations were normalized to the total deconvoluted peak intensity and plotted by clone number and day, allowing for identification of abundant mispairs and half-antibodies (see legend to associate color bars with species) as well as clone selection across collected time points. (*B*) Comparison between clones of correctly paired bispecific antibody as percent total antibody detected at 0, 7, 10, and 14 d.

We then analyzed 72 samples from another set of two transfection pools and 16 single cell line clones (four time points each) and characterized the different species identified (Fig. 3*A*). Data from day 14 of clones 7 and 12 were discarded due to contamination of the clones during growth. As observed with the aforementioned eight pools, a general trend in ion abundance was observed at later culture time points (Fig. 3). Using the RF-MS assay, we identified clones 5 and 11 as the clones providing the greatest potential for correct pair matching for BsIgG. It should be noted that the correct pair match (Fig. 3*A* red bars) is also isobaric to an antibody species with both light chains mispaired. Generally, LC mispairing is driven by chain expression stoichiometry mismatches, so the levels of double mispaired LC is significantly lower than the single mispaired LC. The levels of double mispaired LC can be estimated by the abundance of single mispaired species through a validated probability-based mathematical model to differentiate the two isobaric species (26). In addition, for differentiating the isobaric species on our lead clones, we also perform MS characterization using middle down digestion to analyze the individual Fab arms (23).

It should be noted that differences in ionization efficiency between antibody species produces MS-response biases, creating difficulties in absolute quantitation comparison between different antibody species. However, for our application of relative quantitating the same species among different spectra sufficiently allows for ranking of cell line clones by their production of correctly paired bispecific antibodies. Comparing the RF-MS results from 62 single cell line clones at day 14 to the LC-MS results ran in an independent laboratory for percent of correctly paired BsIgG led to a correlation with R square = 0.9 for the best clones (*SI Appendix*, Fig. S8). Since the 248 samples were analyzed in 2 h, the throughput of RF-MS versus conventional LC-MS was increased by nearly 10-fold without compromising data quality.

### Qualifying the RF-MS Platform for Analyzing Complex Plasma Samples.

**Fig. 4.**
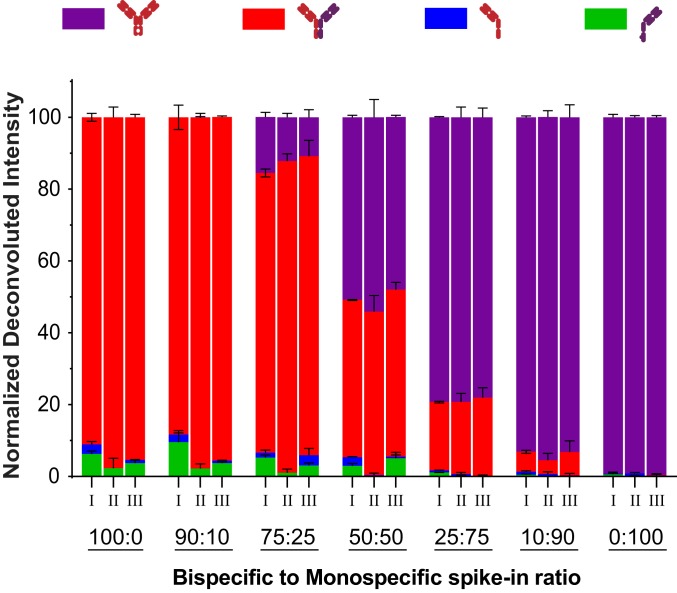
Rapidfire mass spectrometry is applicable for screening antibodies from matrixes as complex as serum. Composition analysis revealed the measured versus experimental composition ratios derived from normalized deconvoluted intensities for the spiked-in samples in buffer (I) and serum (II) after undergoing affinity capture. For buffer, samples without affinity capture were also compared for purification bias (III). Half-antibody impurities (blue and green bars) <5% were detected from the purified bispecific stocks. Since those half-antibodies were associated with the bispecific antibody samples, they were included in the correct bispecific composition for calculation purposes. The purple and red bar correspond to the composition detected, respectively, as monospecific and bispecific antibodies.

While the cell media does contain ion suppressing agents, it is apparent that the complexity is far less than that of other biological matrices. In order to expand the utility of our newly established platform, we evaluated the feasibility to screen for bispecific antibody impurities from matrices as complex as serum by analyzing monospecific and bispecific antibodies spiked in various ratios. The purpose of spiking in monospecific antibodies is to mimic the undesirable homodimeric impurity. Comparing the spiked in composition ratios versus the measured ratios, derived from normalized deconvoluted intensities for the buffer samples with and without affinity capture (Fig. 4), an average accuracy of 6% and 13% composition, respectively, was observed for all concentrations tested. This suggests a low bias in the affinity capture for the two forms tested in complex plasma matrix. Similarly, an average accuracy of 10% was achieved after undergoing the RF-MS workflow, suggesting that the purification step for the analytes tested was highly specific with little bias attributed to sample complexity (Figs. 2 and 4). An average precision of CV 12% was obtained for all samples measured, suggesting that the screening platform is highly repeatable.

### Summary.

We present a high-throughput ESI-MS strategy for qualitative and quantitative analyses of antibodies from a complex mixture with 10-fold throughput enhancement using RF-MS. The utility can be expanded to many other applications that can exploit the advantages of high-throughput intact protein analyses including but not limited to pharmacokinetic analyses, enzymatic screening, biotransformation characterization, and quality control screening. Future work in our laboratory will involve utilizing automation of the affinity capture procedure to enable screening of thousands of samples from complex mixtures.

## Materials and Methods

### Sample Preparation for RapidFire-TOF and LC-MS Analysis.

Chinese hamster ovary cells were cultured in a proprietary Dulbecco's modified Eagle medium/F12-based medium in shake flask vessels at 37 °C and 5% CO_2_. Cells were passaged with a seeding density of 3 × 10^5^/mL, every 3 to 4 d. Platform fed-batch production was performed in shake flasks with proprietary chemically defined media together with bolus feeds on days 3, 7, and 10 with a temperature to 35 °C on day 3. Harvested cell culture fluid (HCCF) from 8 BsIgG-producing cell pools and 62 BsIgG-producing single cell clones were collected either from seed train culture or fed-batch production for RapidFire-TOF analysis. Spiked in samples of the control, monospecific and bispecific antibodies were mixed in at different indicated ratios in buffer (25 mM Tris, 25 mM sodium chloride, pH 7) and Sprague-Dawley rat plasma at a final total concentration of 100 µg/mL. The samples were then subjected to affinity capture followed by RapidFire-TOF analysis.

### Protein A Affinity Capture and Elution.

All reagents prior to the elution step were contained in Thermo Scientific Kingfisher 96-deep–well, V-bottomed polypropylene blocks. Elution was carried out in Thermo Scientific Kingfisher 96-well, V-bottomed polypropylene plates. All bead-handling steps were performed by a Thermo Scientific Kingfisher 96 instrument. Bispecific antibody samples in cell growth media and spiked in samples were diluted 1:3 or 1:8, respectively, in phosphate-buffered saline (PBS) to a final volume of 400 µL prior to capture. Magnetic Protein A Dynabeads (Invitrogen) were diluted to 3.75 mg/mL in PBS and washed twice in PBS prior to introducing bispecific antibody samples. Beads were incubated and agitated with samples for 2 h at room temperature. After incubation, sample-bound beads were washed twice with PBS and three times with LC-MS grade water before elution in 75 to 100 µL of 1% formic acid, 30% acetonitrile. Beads were then removed and plates were heat-sealed with aluminum seals. Sealed plates were stored at 4 °C until analysis.

### RapidFire-MS Analysis.

Unless indicated, all analyses were carried out on an Agilent RapidFire 365 coupled to an Agilent 6230 TOF mass spectrometer with a Dual Agilent Jet Stream electrospray ionization source. All RapidFire steps were carried out at room temperature. RapidFire pump 1 supplied loading buffer (0.1% formic acid, 10% acetonitrile) at 200 µL/min. RapidFire pumps 2 and 3 supplied wash and elution buffer (0.1% formic acid, 80% acetonitrile) at 600 µL/min and 400 µL/min, respectively. Pump 2 was used to flush the sample loop after the sample was loaded onto the solid-phase extraction (SPE) cartridge. All solvents and solutions used were LC-MS grade. Samples were drawn from the plate into a 10 µL loop prior and loaded onto an Optimize Technologies Opti-Lynx C4 micro trap SPE cartridge (1.0 5.0 mm, 4,000 Å pores). Samples were loaded onto the SPE cartridge in loading buffer over 15 s before elution in elution buffer over 8 s into the TOF-MS, which operated in positive ionization mode and acquired mass range from 200 to 3,200 mass-to-charge ratio (*m/z*) set in extended dynamic range (2 GHz) at a rate of 1 spectrum/s. Equilibration of the cartridge in loading buffer occurred for 700 ms. For analyses that were performed using Fourier transform mass spectrometry, the RapidFire supplied an elution flow rate 100 μL/min over 30 s, coupled to Orbitrap Exploris 480 (Thermo Scientific). The spectra were acquired in positive ionization mode from 1,500 to 4,000 *m/z*, SID 100 V, automatic gain control target 1 × 10^6^ charges, resolution 15,000 at 400 *m/z*, with higher-energy collisional dissociation collisional cooling at high pressure in intact protein mode.

### Data Analysis.

Deconvolution of raw spectra within the elution time window was performed using either Agilent MassHunter BioConfirm or Protein Metrics Intact Mass (v3.2.34). Orbitrap data were analyzed using Xcalibur v4.2. Possible bispecific antibody pairings were identified by their average theoretical masses. Relative abundance of each bispecific antibody pairing and half-antibody was calculated by dividing its deconvoluted MS signal intensity by the total intensity of the observed bispecific antibody and half-antibody species.

### Data Availability.

All data are included in the manuscript and *SI Appendix*.

## Supplementary Material

Supplementary File
