# Supplementary material for: High-throughput antibody screening from complex matrices using intact protein electrospray mass spectrometry
Source: Proc Natl Acad Sci U S A. 2020 Apr 23;117(18):9851–6. doi: 10.1073/pnas.1917383117 (PMC7211930; doi:10.1073/pnas.1917383117)
Supplement: Supplementary File [file pnas.1917383117.sapp.pdf]

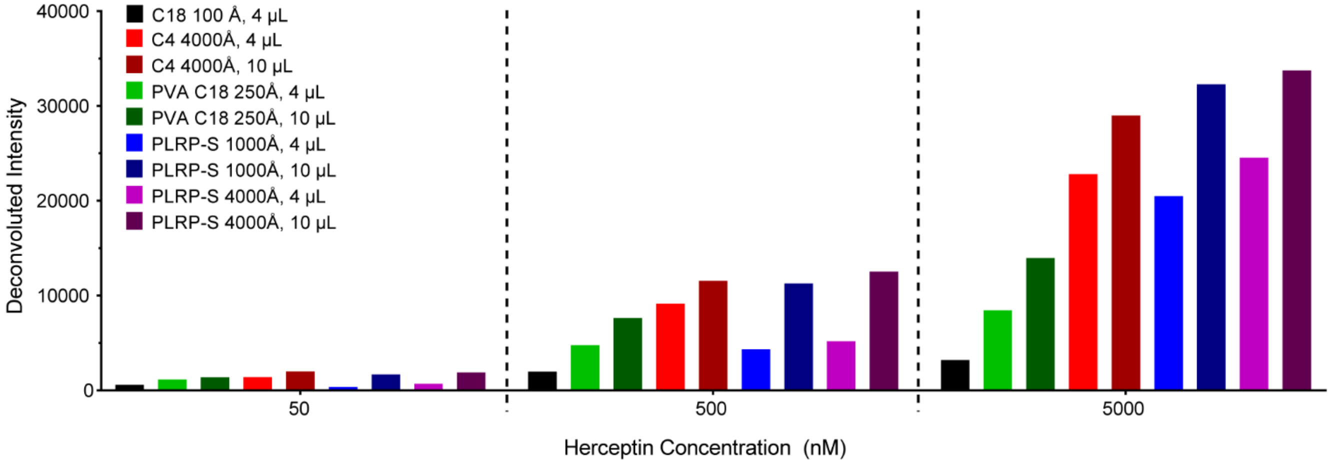

Supplementary Fig. 1 | Comparison of SPE cartridge resins for RapidFire-TOF analysis of trastuzumab at 50, 500, and 5000 nM (n = 3 for each resin).

www.pnas.org/cgi/doi/10.1073/pnas.1917383117

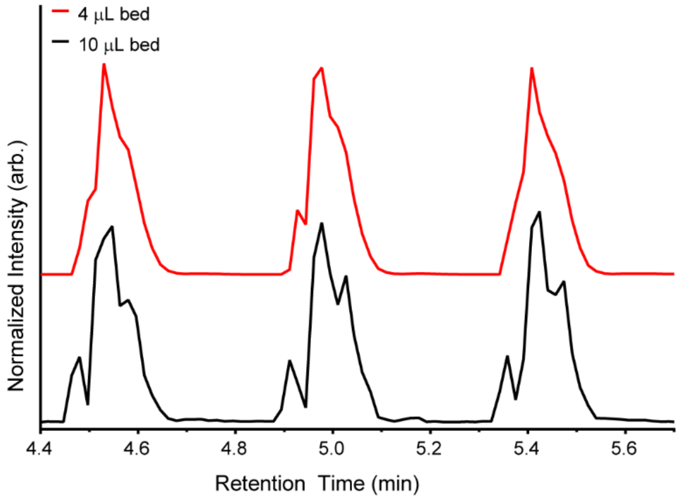

Supplementary Fig. 2 | Comparison of 4 µL and 10 µL bed volumes for 4000 Å C4 SPE cartridges. Pronounced peak-splitting was observed for 10 µL bed volumes.

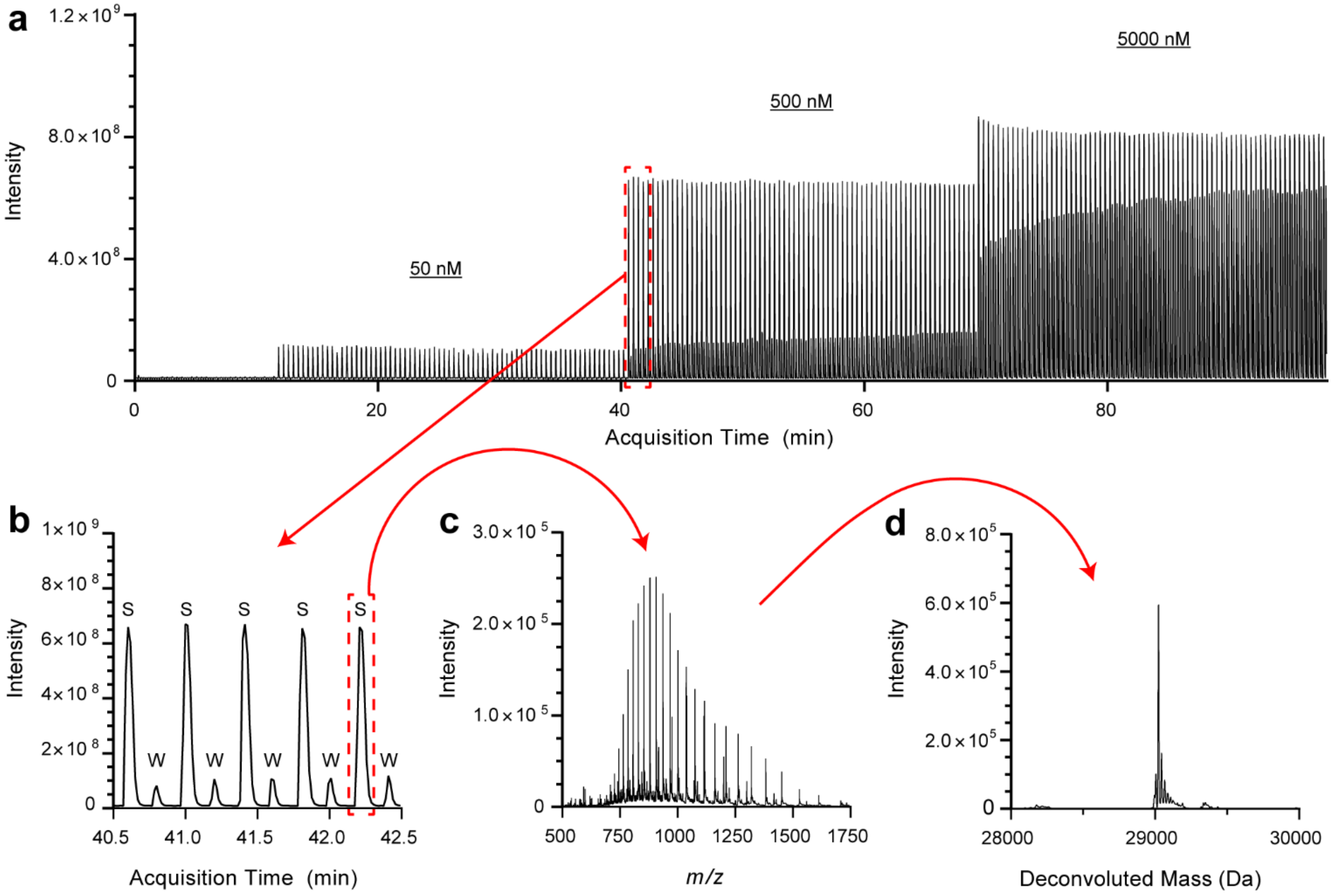

Supplementary Fig. 3 | (**a**) Carbonic anhydrase (~29 kDa protein) ionized efficiently between 10-5000 nM (displayed 50, 500, and 5000 nM extracted ion chromatogram (EIC) extracted over a 600-1600 *m/z* range). Each peak of the chromatogram corresponds to an injection from the RapidFire to the TOF. (**b**) Zoomed-in region of carbonic anhydrase EIC marked in Supplementary Fig. 3a. Peaks labeled with "S" are sample injections, peaks labeled with "W" are wash injections. (**c**) Raw mass spectrum of a sample peak selected in Supplementary Fig. 3b. (**d**) Deconvoluted spectra of raw mass spectrum shown in Supplementary Fig. 3c.

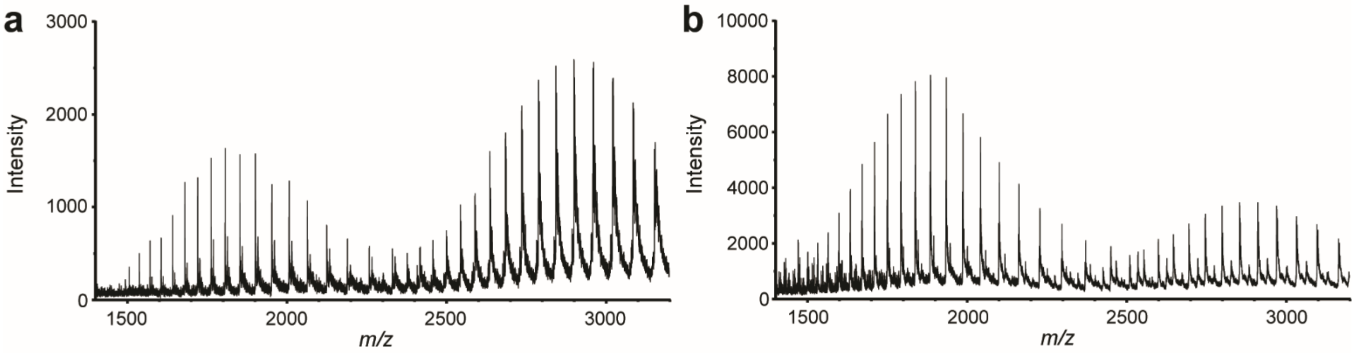

Supplementary Fig. 4 | (**a**) Mass spectrum of protein A captured bispecific antibody at 10 µg/mL. (**b**) Mass spectrum of protein A captured bispecific antibody from cell culture seed train (Day 0). Comparison of spectral intensities between protein A captured bispecific antibody at 10 µg/mL and cell culture seed train material indicates cell culture samples are above limit of detection.

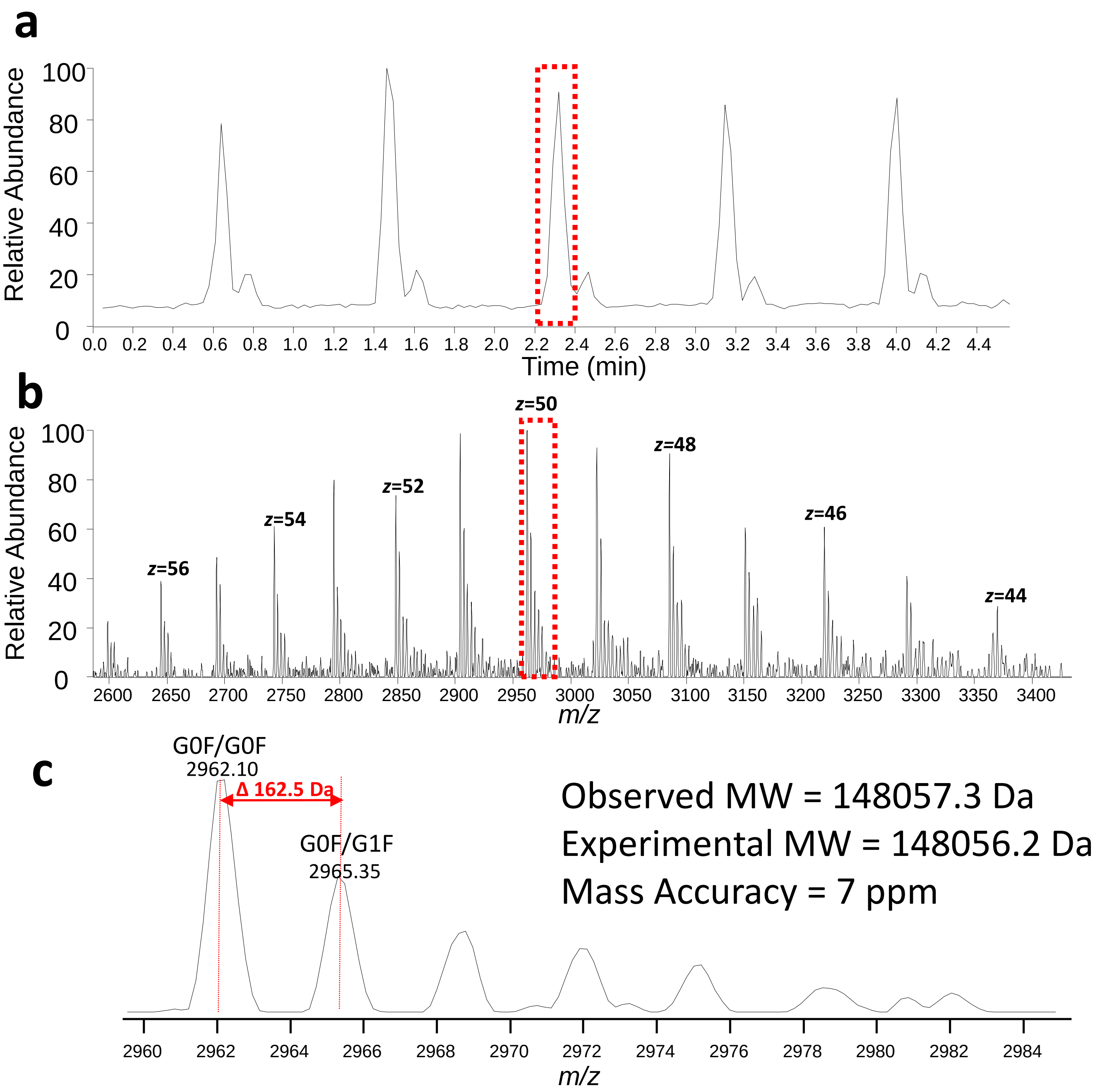

Supplementary Fig. 5 | (**a**) Base peak chromatogram showing five injections of 1 µg/mL trastuzumab from RF coupled to high resolution Orbitrap MS. (**b**) Raw mass spectrum of the charge state envelope of the highlighted averaged scans highlighted in Supplementary Fig. 5a. (**c**) Zoomed in spectrum of the charge state z = 50 peaks shows the baseline separation resolution of the different glycoforms attained. Mass deconvolution of the charge state envelope revealed an observed MW for the trastuzumab glycoform G0F/G0F within 7 ppm mass accuracy.

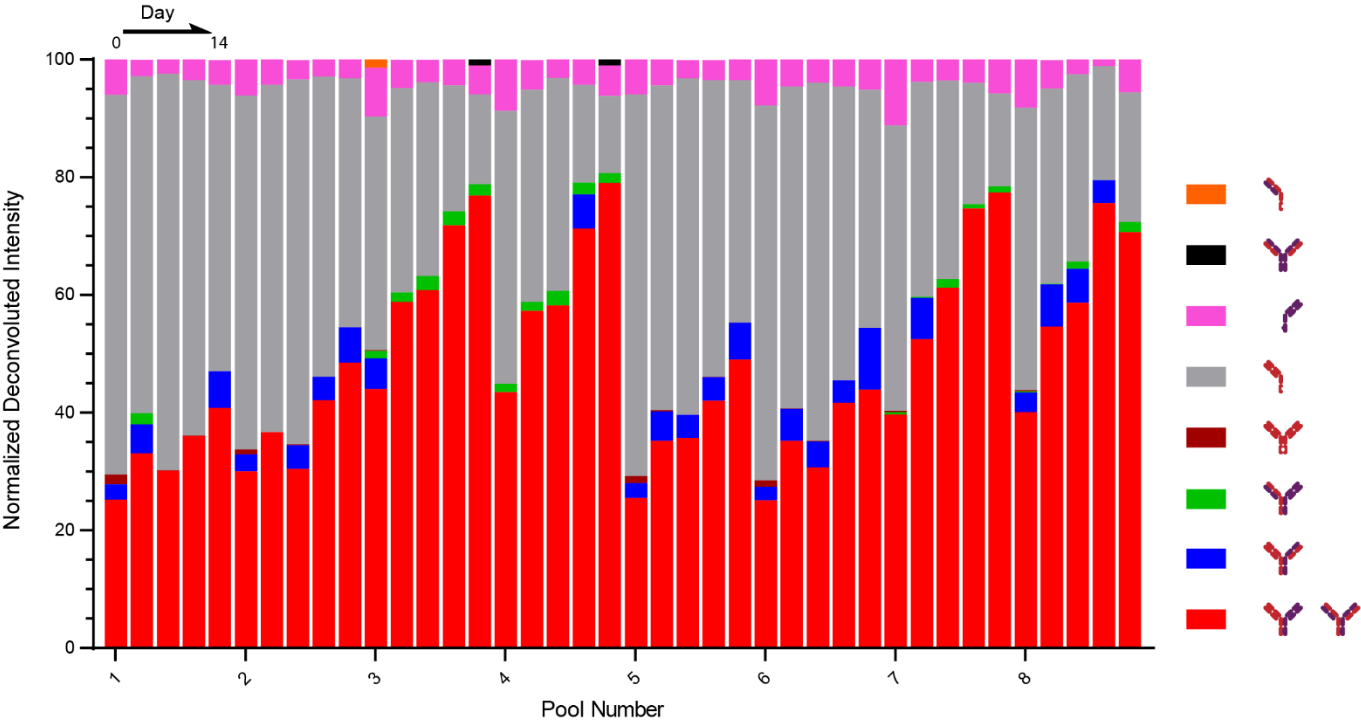

Supplementary Fig. 6 | Eight different BsIgG-producing pools collected at days 0, 7, 10, and 14 after protein A affinity capture and RF-TOF analysis. Deconvoluted intensities of the different combinations of bispecific antibody heavy chain and light chain conjugations were normalized to the total deconvoluted peak intensity and plotted by clone number and day, allowing for comparison of BsIgG production efficiency across pools and time-points.

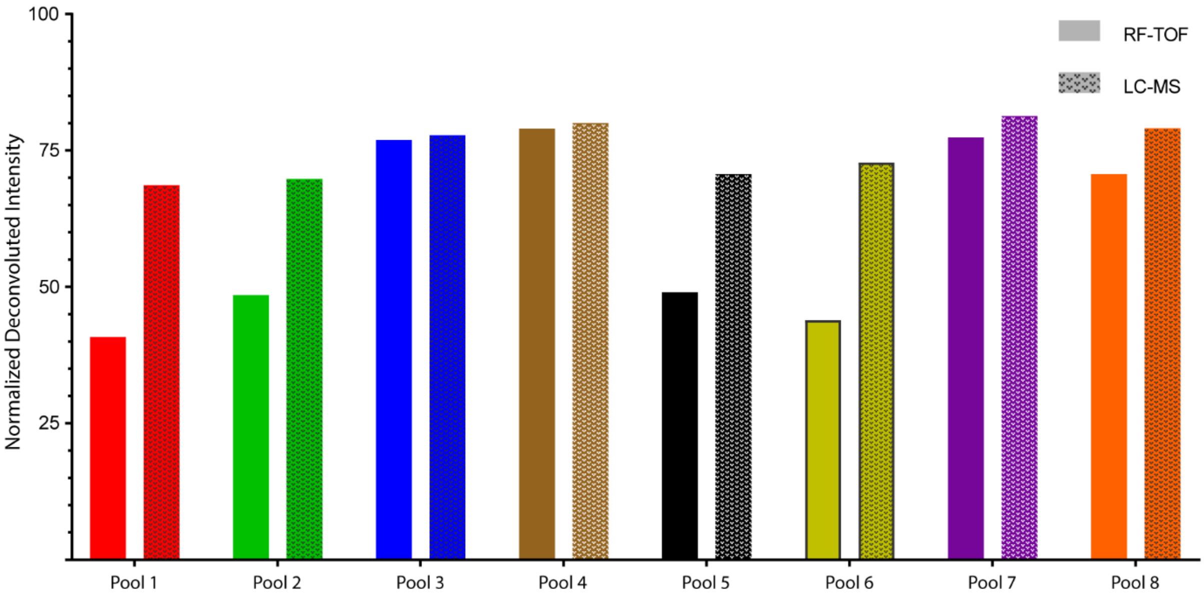

Supplementary Fig. 7 | Comparison of RF-MS data against LC-MS data for 8 pooled clones. Data for the best pools (3, 4, 7, and 8) matched between analytical methods. For the lower producing pools, the LC-MS method yielded a higher percent of correctly matched bispecific antibodies.

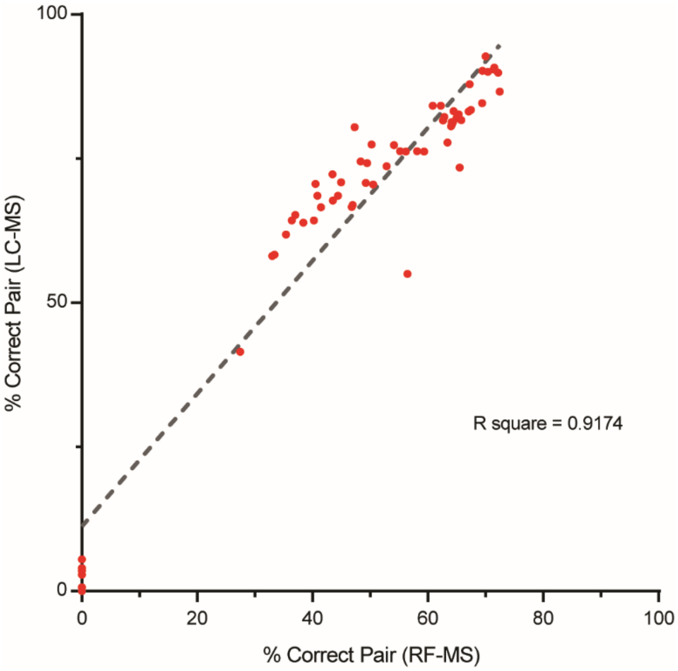

Supplementary Fig. 8 | Correlation plot of percent correctly paired BsIgG between RF-MS and LC-MS data for day 14 samples from 62 clones. Correlation between the two analytical methods yielded an r-square value = 0.9.
